# Sipros Ensemble improves database searching and filtering for complex metaproteomics

**DOI:** 10.1093/bioinformatics/btx601

**Published:** 2017-09-22

**Authors:** Xuan Guo, Zhou Li, Qiuming Yao, Ryan S Mueller, Jimmy K Eng, David L Tabb, William Judson Hervey, Chongle Pan

**Affiliations:** 1 Graduate School of Genome Science and Technology, University of Tennessee, Knoxville, TN, USA; 2 Computer Science and Mathematics Division, Oak Ridge National Laboratory, Oak Ridge, TN, USA; 3 Department of Computer Science and Engineering, University of North Texas, Denton, TX, USA; 4 Department of Microbiology, Oregon State University, Corvallis, OR, USA; 5 Proteomics Resource, University of Washington, Seattle, WA, USA; 6 DST/NRF Centre of Excellence for Biomedical Tuberculosis Research, SAMRC Centre for Tuberculosis Research, Division of Molecular Biology and Human Genetics, Faculty of Medicine and Health Sciences, Stellenbosch University, Cape Town, South Africa; 7 Naval Research Laboratory, Center for Bio/Molecular Science & Engineering (Code 6910), Washington, DC, USA

## Abstract

**Motivation:**

Complex microbial communities can be characterized by metagenomics and metaproteomics. However, metagenome assemblies often generate enormous, and yet incomplete, protein databases, which undermines the identification of peptides and proteins in metaproteomics. This challenge calls for increased discrimination of true identifications from false identifications by database searching and filtering algorithms in metaproteomics.

**Results:**

Sipros Ensemble was developed here for metaproteomics using an ensemble approach. Three diverse scoring functions from MyriMatch, Comet and the original Sipros were incorporated within a single database searching engine. Supervised classification with logistic regression was used to filter database searching results. Benchmarking with soil and marine microbial communities demonstrated a higher number of peptide and protein identifications by Sipros Ensemble than MyriMatch/Percolator, Comet/Percolator, MS-GF+/Percolator, Comet & MyriMatch/iProphet and Comet & MyriMatch & MS-GF+/iProphet. Sipros Ensemble was computationally efficient and scalable on supercomputers.

**Availability and implementation:**

Freely available under the GNU GPL license at http://sipros.omicsbio.org.

**Supplementary information:**

Supplementary data are available at *Bioinformatics* online.

## 1 Introduction

Microbial communities drive nutrient cycling in aquatic and terrestrial ecosystems and influence the health of human, animal and plant hosts. The metabolic activities of a microbial community can be inferred from the proteomes of its constituent microorganisms. In a typical metaproteomics experiment, total proteins are extracted from environmental samples of a microbial community and then measured by liquid chromatography-tandem mass spectrometry (LC-MS/MS) using a ‘shotgun’ proteomics approach (Washburn *et al.*, 2001). All acquired tandem mass spectra (MS2) are compared with predicted peptides from a protein sequence database in a computational procedure called database searching (Chatterjee *et al.*, 2016; Eng *et al.*, 2011; Sadygov *et al.*, 2004; Xiong *et al.*, 2015). A statistically significant match between a peptide and an MS2 spectrum, referred to as a peptide-spectrum match (PSM), provides an identification of the peptide by this spectrum and an identification of the protein containing this peptide. Reversed protein sequences are often added to a protein database as decoys to estimate the false discovery rates (FDR) of peptide and protein identifications (Elias and Gygi, 2007; Peng *et al.*, 2003). PSMs are typically filtered with a score threshold to generate a set of confident PSMs at a specific FDR.

Database searching can provide comprehensive and confident identifications of proteins in single-organism proteomics ranging from axenic bacterial cultures to human tissue samples. The protein databases of these organisms contain only thousands or tens of thousands of protein sequences and provide relatively complete representations of the actual proteins in their proteome samples. However, database searching presents uniquely formidable computational challenges for metaproteomics of microbial communities (Xiong *et al.*, 2015). The metaproteomic protein databases constructed *in silico* from metagenome assemblies may contain millions of predicted proteins spanning thousands of organisms in complex communities (Ahn *et al.*, 2014; Haider *et al.*, 2014). This requires the scoring function of a database searching algorithm to evaluate up to hundreds of times more peptide candidates in community metaproteomics than single-organism proteomics (Chatterjee *et al.*, 2016). While an MS/MS spectrum should have a high score for its match with the correct peptide, the scores of the random matches generally follow a probabilistic distribution with a small tail towards high scores. Therefore, as databases of candidate peptides increase in size, the probability of an incorrect random match that scores higher than the correct match for a spectrum increases as well. Furthermore, because of the incomplete assembly of metagenomes and potential technical biases in sample extractions for metagenomics and metaproteomics, the large protein databases used in metaproteomics are still incomplete and biased representations of the actual proteins in metaproteome samples. As a result, a metaproteomics measurement often contains many spectra originating from peptides not included in these incomplete protein databases. These spectra have no true PSMs to out-rank their high-scoring false, random PSMs. To filter out the high-scoring false PSMs and control the FDR of identifications, a database searching algorithm then needs to set a score threshold for metaproteomics higher than single-organisms proteomics, resulting in the loss of many true PSMs scored below this stringent threshold. These computational challenges often lead to a much smaller number of peptide and protein identifications in metaproteomics analyses of complex communities than comparable proteomics analyses of single organisms.

In this study, we developed a general database searching and filtering algorithm, Sipros Ensemble, for shotgun proteomics analysis of single organisms and microbial communities. It was optimized for metaproteomics to address the computational challenges described above. Two key innovations in Sipros Ensemble were the integration of three diverse existing scoring functions into a single database searching engine and the formulation of PSM filtering as a supervised classification problem. These features enabled Sipros Ensemble to produce substantially higher numbers of peptide and protein identifications in complex metaproteomics datasets than the existing database searching and filtering algorithms benchmarked here.

## 2 Algorithm and implementation

### 2.1 Ensemble searching in Sipros

Sipros Ensemble searches all MS2 spectra against a protein database that contains both target protein sequences and reversed sequences of target proteins as decoys. The database searching iterates between a peptide generation module and a peptide scoring module as the original Sipros (Hyatt and Pan, 2012). The multi-threading parallelism was re-implemented using a producer-consumer model provided by the tasking function in OpenMP 3.0. A single thread serves as the producer of tasks and executes the peptide generation module, which digests proteins to peptides, then matches peptides with spectra by precursor masses to generate PSMs, and finally packages PSMs into tasks (default: 20 000 PSMs per task). All the remaining threads serve as the consumers of tasks and run the peptide scoring module to score PSMs. The producer-consumer tasking parallelism in Sipros Ensemble provided better multi-threading scalability than the simple spectrum-level parallelism in the original Sipros (data not shown).

The peptide scoring module of Sipros Ensemble incorporates the multivariate hypergeometric scoring function (MVH) from the MyriMatch algorithm (Tabb *et al.*, 2007), the cross-correlation scoring function (Xcorr) (Eng *et al.*, 1994) from the Comet algorithm (Eng *et al.*, 2013), and the weighted dot product scoring function (WDP) from the original Sipros algorithm (Pan *et al.*, 2011; Wang *et al.*, 2013). Only C ++ codes for the MVH scoring from MyriMatch (∼750 lines out of ∼17 000 lines in the MyriMatch codebase) and only C ++ codes for the Xcorr scoring from Comet (∼1450 lines out of ∼17 200 lines in the Comet codebase) were integrated into the Sipros C ++ codebase (a total of ∼7800 lines). The other functionalities of these two algorithms, including the mzFidelity scoring in MyriMatch and the expectation value scoring in Comet, were not incorporated in Sipros Ensemble. For most PSMs, Sipros Ensemble generated the same MVH, Xcorr and WDP scores as the original algorithms (data not shown).

Because MVH is more memory-efficient than Xcorr and more CPU-efficient than WDP, MVH is used as the first scoring function in Sipros Ensemble. For each MS2 spectrum, all peptide candidates are first scored by MVH and the top-50 candidates ranked by MVH are then scored by Xcorr and WDP. Scoring top-200 MVH candidates by Xcorr and WDP led to ∼6% higher peak memory usage and ∼13% longer wall-clock time, but virtually no difference in the identification results.

Comet has a relatively high memory usage because it saves all spectra in memory using a sparse matrix representation to speed up the Xcorr calculation. To reduce memory usage, each thread in Sipros Ensemble converts a spectrum to a sparse matrix on the fly, calculates the Xcorr scores for all top candidates of this spectrum, and deletes the sparse matrix before scoring the next spectrum.

Sipros Ensemble ranks the top-50 peptides by each scoring function and outputs the union of top-5 peptides by MVH, top-5 peptides by Xcorr and top-5 peptides by WDP. The scoring results of these peptides can be printed out in a custom tab-delimited format for filtering by Sipros Ensemble or in the pepXML format for third-party filtering algorithms.

### 2.2 Ensemble filtering in Sipros

To filter the database searching results, Sipros Ensemble calculates the following 10 features for every PSM.

- MVH: MVH score;
- Xcorr: Xcorr score;
- WDP: WDP score;
- ΔMVH: score differential for the MVH score by Equation 1 below;
- ΔXcorr: score differential for the Xcorr score by Equation 1 below;
- ΔWDP: score differential for the WDP score by Equation 1 below;
- ΔM: absolute difference between the calculated mass and the measured mass of the precursor ion;
- #MCS: number of missed cleavage sites in the peptide of the PSM;
- #PEP: spectrum count for the peptide of the PSM, including all modification forms and charge states of the peptide.
- #PRO: spectrum count for the protein or the protein group of the PSM. The spectrum count of a protein or a protein group includes both unique peptides and non-unique peptides. If this PSM can be assigned to multiple proteins or protein groups, the one with the highest spectrum count is used.

The score differential of a PSM for a given scoring function is calculated as:

(1) $$\Delta =\frac{S_{c}-S_{b}}{S_{b}}$$

where $S_{c}$ is the score of this PSM and $S_{b}$ is the highest score of other PSMs for this spectrum. The score differential is positive for the top-ranking PSMs and negative for lower-ranking PSMs. Peptide- and protein-level features, similar to #PEP and #PRO, were also used by Percolator (Käll *et al.*, 2007) and iProphet (Shteynberg *et al.*, 2011). Filtering PSMs with peptide- and protein-level features would make PSM identifications no longer independent events for peptide and protein identifications.

Each scoring function has a top-ranking PSM for a spectrum. A PSM is a unanimous PSM if it is identified as the top-ranking PSM by all three scoring functions. All unanimous PSMs from target proteins are incorporated into the positive training data. The reversed proteins in the database are randomly assigned to a training set for building classifiers and a test set for estimating FDRs. Decoy PSMs from the reversed proteins in the training set are incorporated into the negative training data. The positive and negative training data are used to train the following supervised binary classifiers: logistic regression with L2 regularization, random forest (200 decision trees, Gini impurity, minimum samples of an internal node = 800, and minimum samples of leaf node = 50), AdaBoost (200 estimators), deep learning (three layers, each with 32 perceptrons and sigmoid as the activation function) and stacking (logistic regression as the meta-layer to combine predictions from the previous four models). Logistic regression, random forest and AdaBoost were implemented using the scikit-learn library (Pedregosa *et al.*, 2011). Deep learning was implemented using the Keras library (Chollet, 2015). Default parameters in these libraries were used unless specified above.

A trained classifier is used to evaluate the top-ranking PSM(s) from the three scoring functions for every spectrum in a proteomics run. For a spectrum with two or three top-ranking PSMs identified by different scoring functions, the PSM with the highest classification score is selected as the top-ranking PSM for filtering. Every spectrum has one and only one PSM for filtering based on its classification score. The score threshold is adjusted to reach a user-defined FDR. The FDR of a set of filtered PSM is calculated as

(2) $$FDR=\frac{\#TestDecoy}{\alpha \times \#Target}$$

where #Target is the number of target PSMs, #TestDecoy is the number of decoy PSMs from the reversed proteins in the test set, and α is the fraction of the reversed proteins in the test set out of all reversed proteins. Since the reversed proteins in the training set are not used to estimate FDRs to avoid the training bias, #TestDecoy/α is an estimate of the number of decoy PSMs if all reversed proteins are used for FDR estimation. The value of α is 1/2 when all reversed proteins are randomly assigned to the training set and the test set in equal proportions. The performances of the five classifiers were benchmarked, and the logistic regression classifier was selected for PSM filtering in Sipros Ensemble.

Sipros Ensemble assembles the filtered PSMs to peptides and proteins as described previously (Hyatt and Pan, 2012; Nesvizhskii and Aebersold, 2005; Pan *et al.*, 2011; Wang *et al.*, 2013). A peptide is identified if any of its PSMs is identified. A protein is identified if at least one unique peptide from this protein is identified. These criteria for peptide and protein identifications can be adjusted by users. Proteins with indistinguishable PSMs are aggregated to protein groups. FDRs for identified peptides and proteins are also estimated by Equation 2 using the reversed proteins in the test set. Sipros Ensemble can adjust the score threshold for filtering PSMs to reach a user-defined FDR at the peptide level or the protein level. Sipros Ensemble is freely available at http://sipros.omicsbio.org, including the source code, documentation and benchmarking results.

## 3 Results

### 3.1 Ensemble searching with three diverse scoring functions

The three scoring functions in Sipros Ensemble were compared using three metaproteomes from a soil community (Butterfield *et al.*, 2016) and three metaproteomes from a marine community (Bryson *et al.*, 2016) (Table 1). These metaproteomes were all measured using the Multidimensional Protein Identification Technology (MudPIT) approach (Washburn *et al.*, 2001) on an LTQ Orbitrap Elite mass spectrometer (Thermo Scientific). Their matched metagenomes were used to construct a soil protein databases containing ∼3.4 million target proteins and a marine protein database containing ∼392 000 target proteins. The mass spectrometry data and protein databases are available from the ProteomeXchange Consortium via the PRIDE repository with the dataset identifier of PXD007587. Details on these benchmarking datasets are described in the Supplementary Methods.

**Table 1.** Consistency and accuracy of PSM identifications by three diverse scoring functions

		Soil 1	Soil 2	Soil 3	Marine 1	Marine 2	Marine 3
Total	# Spectra	374 692	454 828	360 409	128 648	132 605	119 403
	% Spectra	100%	100%	100%	100%	100%	100%
Unanimous PSM	# Spectra^a^	126 386	117 693	108 730	54 357	47 759	56 139
	% Spectra^b^	34%	26%	30%	42%	36%	47%
	% Decoy^c^	7%	8%	7%	4%	4%	3%
Majority PSM:	# Spectra	42 721	53 157	43 706	13 343	15 053	12 014
WDP & Xcorr	% Spectra	11%	12%	12%	10%	11%	10%
Minority PSM: MVH	% Decoy, Majority	36%	36%	33%	29%	29%	27%
	% Decoy, Minority	44%	45%	44%	41%	44%	40%
Majority PSM:	# Spectra	25 558	29 713	23 414	8677	8231	7661
WDP & MVH	% Spectra	7%	7%	6%	7%	6%	6%
Minority PSM: Xcorr	% Decoy, Majority	37%	39%	37%	27%	32%	27%
	% Decoy, Minority	43%	44%	42%	41%	42%	40%
Majority PSM:	# Spectra	20 010	26 478	23 053	5353	7445	4836
MVH & Xcorr	% Spectra	5%	6%	6%	4%	6%	4%
Minority PSM: WDP	% Decoy, Majority	33%	32%	27%	30%	27%	31%
	% Decoy, Minority	45%	46%	46%	41%	43%	38%
Discordant PSM	# Spectra	160 017	227 787	161 506	46 918	54 117	38 753
	% Spectra	43%	50%	45%	36%	41%	32%
	% Decoy, WDP	48%	48%	47%	46%	47%	46%
	% Decoy, Xcorr	47%	48%	46%	47%	46%	46%
	% Decoy, MVH	47%	47%	47%	48%	47%	47%

a Number of spectra in a class.

b Percentage of spectra in a class out of all acquired spectra.

c Percentage of decoy PSMs out of all PSMs in a class or a sub-class.

Because MVH, Xcorr and WDP may rank different peptides as the highest-scoring match for an MS2 spectrum, Sipros Ensemble can assign one, two or three PSMs to an MS2 spectrum. Based on the degree of agreement among the three scoring functions, the spectra in a metaproteomics measurement were divided into three classes: unanimous PSMs, majority/minority PSMs and discordant PSMs. A unanimous PSM had the same peptide ranked by all three scoring functions as the best match for a spectrum. On average, 30% of spectra in soil samples and 42% of spectra in marine samples had unanimous PSMs (Table 1). The average percentages of decoy hits in these unanimous PSMs were 7% for soil and 4% for marine. This showed that the accuracy of identification was very high even without any score-based filtering if the three scoring functions can all agree.

On average, 24% of spectra in soil samples and 21% of spectra in marine samples had majority PSMs, which were agreed by two out of the three scoring functions. The other dissenting scoring function provided minority PSMs for these spectra. These majority/minority PSMs can be further divided into three sub-classes based on the dissenting scoring functions (Table 1). The majority PSMs in soil samples had an average of 34% decoy hits. There was no significant difference in the decoy percentage among the three sub-classes of majority PSMs. The minority PSMs in the three sub-classes all had close to 45% decoy hits, which was ∼5% better than random guesses since the protein databases contained the same number of target and decoy proteins. The much higher percentages of decoys in majority PSMs than unanimous PSMs suggests that the disagreement from one scoring function, no matter which one, was an indicator for the low identification confidence of the majority PSMs agreed on by the other two scoring functions.

The three scoring functions identified three different PSMs for each spectrum in the remaining 46% of spectra in soil samples and 37% of spectra in marine samples. These PSMs were referred to as discordant PSMs. The percentages of decoy hits were calculated separately for the PSMs selected by each scoring function, which were ∼47% on average and ∼3% better than random guesses. Some of these discordant spectra likely originated from peptides whose sequences were not represented in the protein databases. This would result in the ranking of peptide candidates based on divergent trivial preferences of the three scoring functions.

Overall, the results for the different classes of PSMs in Table 1 indicated a low degree of correlation among decoy PSMs and a high degree of agreement among target PSMs identified by the three scoring functions. Using three scoring functions provided significantly better discrimination of target PSMs from decoy PSMs than using only two scoring functions. For example, if only Xcorr and MVH were considered in soil 1, the majority PSMs of MVH and Xcorr would become unanimous PSMs, which would increase the number of decoys in the unanimous PSM class by 75% from 8847 to 15 450. This would also turn the majority PSMs of WDP and Xcorr and the majority PSMs of WDP and MVH into discordant PSMs. Such loss of discriminatory information from leaving out a third scoring function was generally consistent across samples and scoring functions.

### 3.2 Ensemble filtering with supervised classification

After ensemble searching, Sipros Ensemble extracts 10 features on the obtained PSMs for ensemble filtering. Each scoring function provides a score and a score differential as two PSM features. A confident PSM should have three high scores and three large positive score differentials. The MS1 analysis and the proteolysis provide the mass errors of precursor ions and the numbers of missed cleavage sites, respectively, as two features of PSMs. The target PSMs had lower mass errors and less missed cleavage sites than the decoy PSMs (Supplementary Fig. S1). The peptide and protein of a PSM can be identified by other PSMs before filtering. The spectrum counts of peptides and proteins before filtering were also included as two features of PSMs. The target PSMs had higher peptide and protein spectrum counts than the decoy PSMs (Supplementary Fig. S1). We also tested additional features, including precursor charge states, mass windows, peptide length and others used in Percolator (Käll *et al.*, 2007), iProphet (Shteynberg *et al.*, 2011) and PepArML (Edwards, 2013). These features were not used by Sipros Ensemble in production because the filtering results were not improved by including these additional features.

Because of the low percentage of decoys before filtering in the unanimous class, all target unanimous PSMs were incorporated into the positive training data. Decoy PSMs from the reversed proteins assigned to the training set were incorporated into the negative training data. The positive and negative training data were used to train supervised classifiers constructed with the above 10 features of PSMs based on logistic regression, random forest, AdaBoost, deep learning and stacking. Supplementary Table S1 shows the parameters of the 10 features in these classifiers. The PSM identification results at the 1% test FDR were compared between the five classifiers in Supplementary Table S2. Logistic regression generated the highest number of PSM identifications for five out of the six metaproteomes. The FDR training biases were calculated as the differences between the training FDRs estimated using reversed proteins in the training set and the test FDRs estimated using reversed proteins in the test set. Logistic regression had the lower FDR training bias than the other classification algorithms, which may reflect less overfitting of the training data. Because of the high PSM numbers, the low FDR training biases, and the short computing time for training, logistic regression was used in Sipros Ensemble for filtering PSMs.

On average, 77% of the unanimous PSMs, 22% of the majority PSMs, 7% of the minority PSMs and 4% of the discordant PSMs passed the filtering (Supplementary Table S3). This indicates that the filtering algorithm needed to discard many unanimous forward PSMs to reduce FDRs, but was able to recover small fractions of forward PSMs from the majority/minority and discordant classes.

Furthermore, we tested a secondary filtering using the differences between the measured and predicted retention times of PSMs. This was not used in production due to the limited performance gain (Supplementary Results).

### 3.3 Performance comparison of Sipros Ensemble with existing algorithms

Sipros Ensemble was compared with seven different combinations of existing database searching and filtering algorithms on the six soil and marine samples (Table 2 and Supplementary Figs S3–S5). Table 2 shows the identifications of PSMs, peptides and proteins filtered at 1% FDR estimated using the reversed proteins in the test set which was held out from all filtering algorithms. Details on the execution of these algorithms are described in the Supplementary Methods. Percolator has been shown to provide excellent performance with Comet (Park *et al.*, 2008) and MS-GF+ (Granholm *et al.*, 2013). For comparison, Percolator was also used here to filter the MyriMatch results and the WDP scoring results from Sipros Ensemble without Xcorr or MVH. Among the four individual algorithms tested with Percolator, WDP generally provided the best PSM and peptide identification results, and MS-GF+ or Comet generally provided the best protein identification results. The features from MyriMatch and WDP were not optimized for Percolator in this study to the same extent as the previous studies (Granholm *et al.*, 2013; Park *et al.*, 2008), which focused on combining a specific database searching algorithm with Percolator. Thus, the performance of MyriMatch and WDP may not represent their best achievable using Percolator. iProphet from Trans Proteomic Pipeline (TPP) was used with two combinations of Comet, MyriMatch and MS-GF+. The combined search results of these three algorithms were also filtered using the ensemble filtering algorithm in Sipros Ensemble without extensive feature optimization.

**Table 2.** Benchmarking of identification performance using six real-world metaproteomes

Metaproteomes		Soil 1	Soil 2	Soil 3	Marine 1	Marine 2	Marine 3
Search^a^	Filter^b^	# PSM Identifications at PSM FDR 1%^c^					
W	P	102 664	95 009	88 686	46 010	36 999	48 232
M	P	87 328	74 647	69 213	39 576	26 249	41 465
C	P	100 683	92 596	94 842	35 012	32 580	39 923
G	P	97 702	94 341	94 373	36 328	33 241	40 220
C&M	I	127 582	121 166	121 567	49 262	42 688	52 154
C&M&G	I	**130 601**	**124 965**	**125 293**	**54 625**	**48 916**	**57 347**
C&M&G	SE-F	96 220	99 579	90 507	42 811	40 282	46 603
SE-S	SE-F	136 468	125 297	129 732	56 170	51 438	58 870
Search^a^	Filter^b^	# Peptide Identifications at Peptide FDR 1%^c^					
W	P	34 049	31 233	25 618	**28 619**	25 868	**31 708**
M	P	27 700	24 236	20 210	23 572	17 935	26 277
C	P	30 165	27 165	23 680	21 726	22 823	26 173
G	P	30 465	28 693	24 100	21 603	22 252	25 338
C&M	I	35 303	32 594	27 557	27 154	27 403	30 948
C&M&G	I	**36 325**	**33 744**	**28 677**	27 412	**27 990**	31 244
C&M&G	SE-F	30 201	29 179	23 574	26 158	26 597	29 706
SE-S	SE-F	43 914	40 287	35 013	34 576	35 479	38 451
Search^a^	Filter^b^	# Protein Identifications at Protein FDR 1%^c^					
W	P	6660	5996	4636	**7257**	6173	7982
M	P	7142	6546	5654	6536	5892	7101
C	P	7752	7020	6517	6818	**7532**	**8211**
G	P	**8180**	7138	6623	7086	7360	8067
C&M	I	7103	6738	5929	6107	6622	7019
C&M&G	I	7067	6800	5810	6129	6571	7198
C&M&G	SE-F	7966	**7404**	**6746**	6717	7302	7702
SE-S	SE-F	8868	7456	6979	8129	8430	9234

a Searching algorithms: W, WDP; M, Myrimatch; C, Comet; G, MS-GF+; SE-S, Sipros Ensemble Searching.

b Filtering algorithms: P, Percolator; I, iProphet; SE-F, Sipros Ensemble Filtering.

c The best entry was underlined and the second best was in bold.

Across the six metaproteomes, Sipros Ensemble generated more PSM identifications, more peptide identifications and more protein identifications than any other database searching and filtering algorithms at 1% FDR. The iProphet filtering with a combination of Comet, MyriMatch and MS-GF+ searching provided the next best PSM identification results after Sipros Ensemble. The iProphet filtering with a combination of Comet, MyriMatch and MS-GF+ searching or the Percolator filtering with WDP scoring provided the next best peptide identification results after Sipros Ensemble. The Percolator filtering or Sipros Ensemble filtering coupled with Comet, MS-GF+ and WDP provided the next best protein identification results after Sipros Ensemble. The improvement of Sipros Ensemble over the best non-Sipros algorithms was 25% more peptides and 13% more proteins at 1% FDR, for the marine samples on average, and 21% more peptide identifications and 6% more protein identifications at 1% FDR for the soil samples on average.

These algorithms were also compared by searching an *E.coli* proteomics dataset against three protein databases (Table 3). MS-GF+/Percolator identified the most proteins from this dataset at 1% protein FDR using the full *E.coli* database containing concatenated forward-reversed proteins. The target *E.coli* proteins were then randomly sub-sampled at 50% and concatenated with the full target soil database from above and a 10% randomly sub-sampled target soil database. All the target proteins were reversed and added to the databases as decoys for the filtering algorithms. The two synthetic databases simulated the challenges of missing true proteins and overwhelming false proteins in metaproteomics databases. Approximately 35% of spectra in this *E.coli* MS/MS dataset were identified using the two synthetic databases, which was comparable to the spectrum identification rates in the soil and marine datasets. In this *E.coli* proteome sample, only *E.coli* proteins should be true identifications and all non-*E.coli* proteins with no shared peptides with any *E.coli* protein should be false identifications. This enabled filtering the database searching results based on the percentage of non-*E.coli* proteins. Sipros Ensemble identified the most *E.coli* proteins at the 5% non-*E.coli* protein rate for both synthetic databases (Table 3). All algorithms identified fewer proteins from the larger synthetic database than the smaller one. Protein FDRs were also estimated as shown in the parentheses in Table 3 using the test reversed proteins in the two synthetic databases. Some benchmarked algorithms filtered out all test reversed proteins and reached the 0% lower bound for FDR estimation, but still falsely identified 5% non-*E.coli* proteins. But Sipros Ensemble and WDP/Percolator retained substantial numbers of reversed proteins in line with the presence of 5% non-*E.coli* proteins, which indicated their lower training biases and lower FDR estimation errors based on reversed proteins.

**Table 3.** Benchmarking of identification performance using *E.coli* and synthetic metaproteome databases

Databases		100% *E.coli*	50% *E.coli* + 10% soil	50% *E.coli* + 100% soil
Search^a^	Filter^b^	1% FDR^c^	5% Non-*E.coli* proteins^d,e^	
W	P	2062	955 (0.9%)	**888** (0.7%)
M	P	2153	972 (0.0%)	776 (0.0%)
C	P	**2194**	966 (0.0%)	836 (0.0%)
G	P	2197	**974** (0.0%)	803 (0.0%)
C&M	I	2170	915 (**0.3%**)	807 (0.0%)
C&M&G	I	2182	917 (**0.3%**)	815 (0.0%)
C&M&G	SE-F	2158	854 (0.0%)	726 (0.0%)
SE-S	SE-F	2137	1045 (0.9%)	920 (**0.3%**)

a Searching algorithms: W, WDP; M, Myrimatch; C, Comet; G, MS-GF+; SE-S, Sipros Ensemble Searching.

b Filtering algorithms: P, Percolator; I, iProphet; SE-F, Sipros Ensemble Filtering.

c Number of identified *E.coli* proteins filtered at 1% protein FDR estimated by target-decoy searches.

d Number of identified *E.coli* proteins (and their FDRs estimated by target-decoy searches in parenthesis) filtered at 5% non-*E.coli* proteins.

e The best entry was underlined and the second best was in bold.

**Fig. 1:**
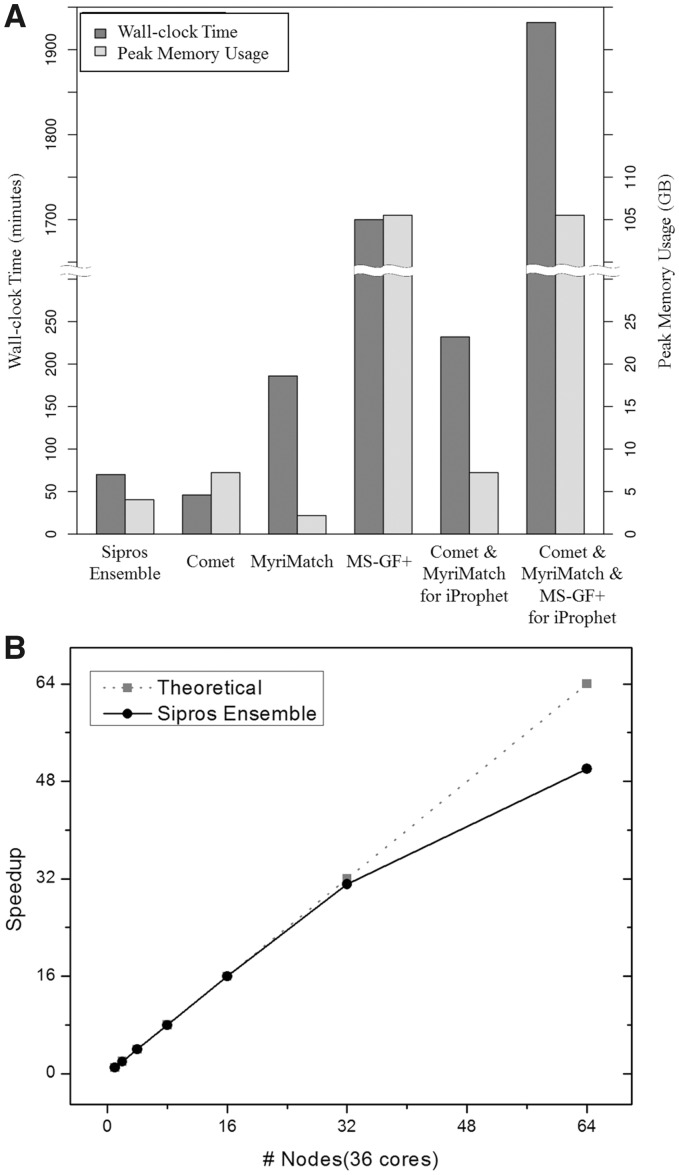
Computational performance of database searching in metaproteomics. (**A**) Comparison of the execution time and peak memory of Sipros Ensemble, Comet, MyriMatch, MS-GF+ and their combinations used by iProphet. (**B**) Computational scalability of Sipros Ensemble on a supercomputer. Only a single LC-MS/MS cycle out of 22 cycles in a MudPIT run was used to measure the computational resources used by the database searching algorithms

To search large databases in complex metaproteomics, a computationally more efficient algorithm should use less CPU time and require a lower amount of physical memory on a computer to accommodate its peak memory footprint during execution. The wall-clock time and the peak memory usage of database searching were compared between these algorithms using the fifth LC-MS/MS cycle out of the 22-cycle MudPIT analysis of soil 1 on a computer with a 16-core Xeon CPU and 128 GB of memory (Fig. 1A). Sipros Ensemble used more wall-clock time, but less peak memory usage, than Comet. Sipros Ensemble used more peak memory, but less wall-clock time, than MyriMatch. MS-GF+ used much more wall-clock time and more peak memory usage than Comet, MyriMatch and Sipros Ensemble. The combination of multiple database searches by iProphet would require the sum of their wall-clock times and the maximum of their peak memory usage. The integration of three scoring functions in Sipros Ensemble used a small fraction of wall-clock time and peak memory usage needed for combining individual database searching algorithms (Fig. 1A). 

The computational architecture of Sipros Ensemble was designed to scale up in supercomputers for PTM identifications (Li *et al.*, 2014, 2017), amino acid mutation detections (Hyatt and Pan, 2012) and proteomic stable isotope probing (Bryson *et al.*, 2016; Marlow *et al.*, 2016; Pan *et al.*, 2011). Scalability of Sipros Ensemble was tested on the Thunder HPC system for the soil 1 metaproteome. Sipros Ensemble achieved close to linear speed-up of its computation using up to 64 compute nodes and 2304 CPU cores (Fig. 1B).

## 4 Discussion

Many existing database searching algorithms incorporate multiple scoring functions. For example, SEQUEST uses the Xcorr scoring function and a preliminary scoring function. X! Tandem generates hyperscore, bscore, yscore and E-value (Fenyo and Beavis, 2003). MyriMatch includes the MVH and mzFidelity score functions. Comet generates Xcorr and expectation values. However, a database searching algorithm typically calculates multiple scores based on similar spectrum preprocessing, fragmentation prediction and scoring statistical models, which results in a high degree of correlation among these scoring functions. To reduce such correlation, an effective approach demonstrated in iProphet (Shteynberg *et al.*, 2011), PepArML (Edwards *et al.*, 2009), MSblender (Kwon *et al.*, 2011) and IPP (Park *et al.*, 2016) was to perform database searching using multiple independent database searching algorithms and combine the results for filtering. However, it is computationally expensive and operationally difficult to search the same dataset with multiple database searching algorithms. Thus, most proteomics studies have been done using only a single database searching algorithm.

In this study, MVH from MyriMatch, Xcorr from Comet and WDP from the original Sipros were integrated into a single database searching algorithm. MyriMatch, Comet and Sipros were used here, because of their excellent performance, compatible C/C ++ code bases, and open-source software licenses. Their scoring results were highly diverse (Table 1). The added computational cost was minimized using a two-tiered scoring strategy pioneered in SEQUEST (Eng *et al.*, 1994) by first scoring all peptide candidates of a spectrum by the CPU- and memory-efficient MVH and then scoring only the top candidates with the other two scoring functions. To the best of our knowledge, this is the first database searching algorithm that has integrated such diverse scoring functions. This showcases the value of collaborative open-source software development.

In previous studies, the parameterization of a statistical model or the training of a machine learning algorithm for PSM filtering was complicated by the fact that, while decoy PSMs must be false PSMs, target PSMs may be true or false PSMs. To solve this problem, Percolator iteratively trains and applies a Support Vector Machine (SVM) classification algorithm, and iProphet also iteratively constructs statistical models using an expectation-maximization algorithm. Here, we observed that the unanimous PSMs supported by all three scoring functions have a very high probability of being true PSMs and, therefore, can be directly used as positive training data. This allowed the formulation of PSM filtering as a straightforward supervised classification problem. Logistic regression was found to provide better performance than other machine learning algorithms for this problem (Supplementary Table S2).

Sipros Ensemble was compared with several combinations of established database searching and filtering algorithms. Sipros Ensemble can be used in general for shotgun proteomics of single organisms and microbial communities. It achieved substantial improvements over existing algorithms for complex metaproteomics as shown here with six real-world metaproteomes (Table 2) and two synthetic metaproteomic databases (Table 3). The higher identification performance of Sipros Ensemble can be attributed to its ability to handle the challenges of missing true proteins and overwhelming false proteins in complex metaproteomics databases. Sipros Ensemble was also computationally efficient and scaled well on high-performance computers (Fig. 1) to reduce the computing time needed for searching large protein databases in metaproteomics.

## Funding

The development of Sipros Ensemble was supported by the Department of Defense's High Performance Computing Modernization Program Application Software Initiative (HASI) under the U.S. Army Corps of Engineers Engineer Research and Development Center (ERDC) by the Department of the Army. A cooperative agreement was executed between the Naval Research Laboratory (NRL-DC) and the University of Tennessee-Knoxville for HASI support. The acquisition of the marine metaproteomics data was supported by the Gordon and Betty Moore Foundation Marine Microbiology Initiative (grant GBMF3302). The acquisition of the soil metaproteomics data was supported by the Office of Science, Office of Biological and Environmental Research, of the US Department of Energy Grant DOE-SC10010566. D.L.T. is funded by a South African MRC grant to Gerhard Walzl for the South African Tuberculosis Bioinformatics Initiative. The opinions and assertions contained herein are those of the authors and are not to be construed as those of the U.S. Navy, the military service at large, or the U.S. Government.

*Conflict of Interest*: none declared.

## Supplementary Material

Supplementary Data
