# Supplementary material for: Sipros Ensemble improves database searching and filtering for complex metaproteomics
Source: Bioinformatics. 2017 Sep 22;34(5):795–802. doi: 10.1093/bioinformatics/btx601 (PMC6192206; doi:10.1093/bioinformatics/btx601)
Supplement: Supplementary Data [file btx601_supp.zip › btx601-suppl_data/Sipros_FigTab_v16_Supplement.pdf]

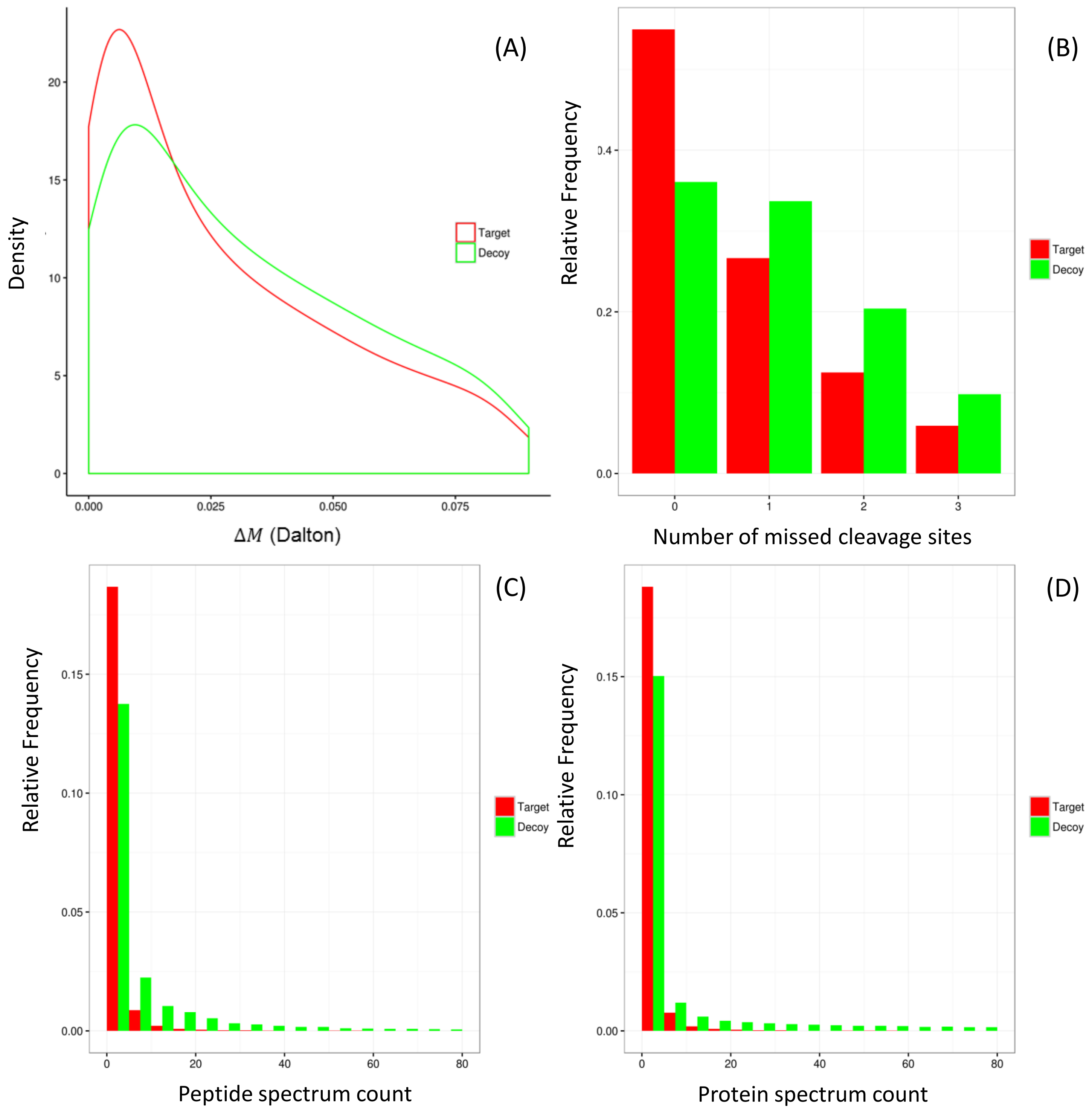

Supplementary Figure 1: Discrimination of target and decoy PSMs by non-MS2-based features. The distributions of the feature values are shown for target PSMs (red) and decoy PSMs (green). A clear separation of the two distributions indicates a discriminatory feature. (A) absolute values of precursor mass errors. (B) numbers of missed cleaved sites. (C) spectrum counts of peptides. (D) spectrum counts of proteins.

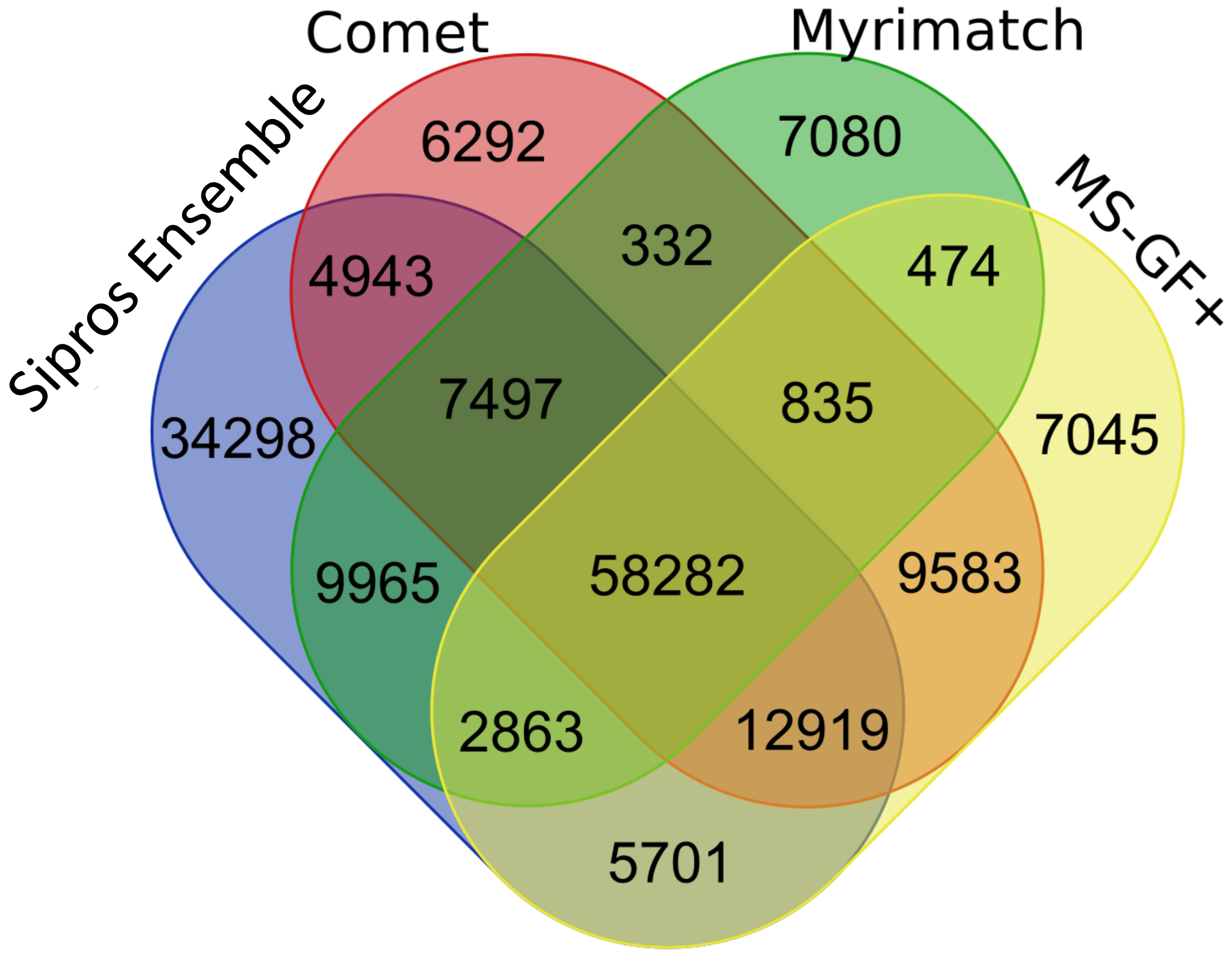

Supplementary Figure 2: Overlap of PSM identifications between Sipros Ensemble and single database searching engines. Comet, MyriMatch and MS-GF+ were all filtered with Percolator. Results were generated using soil 1 at 1% PSM FDR.

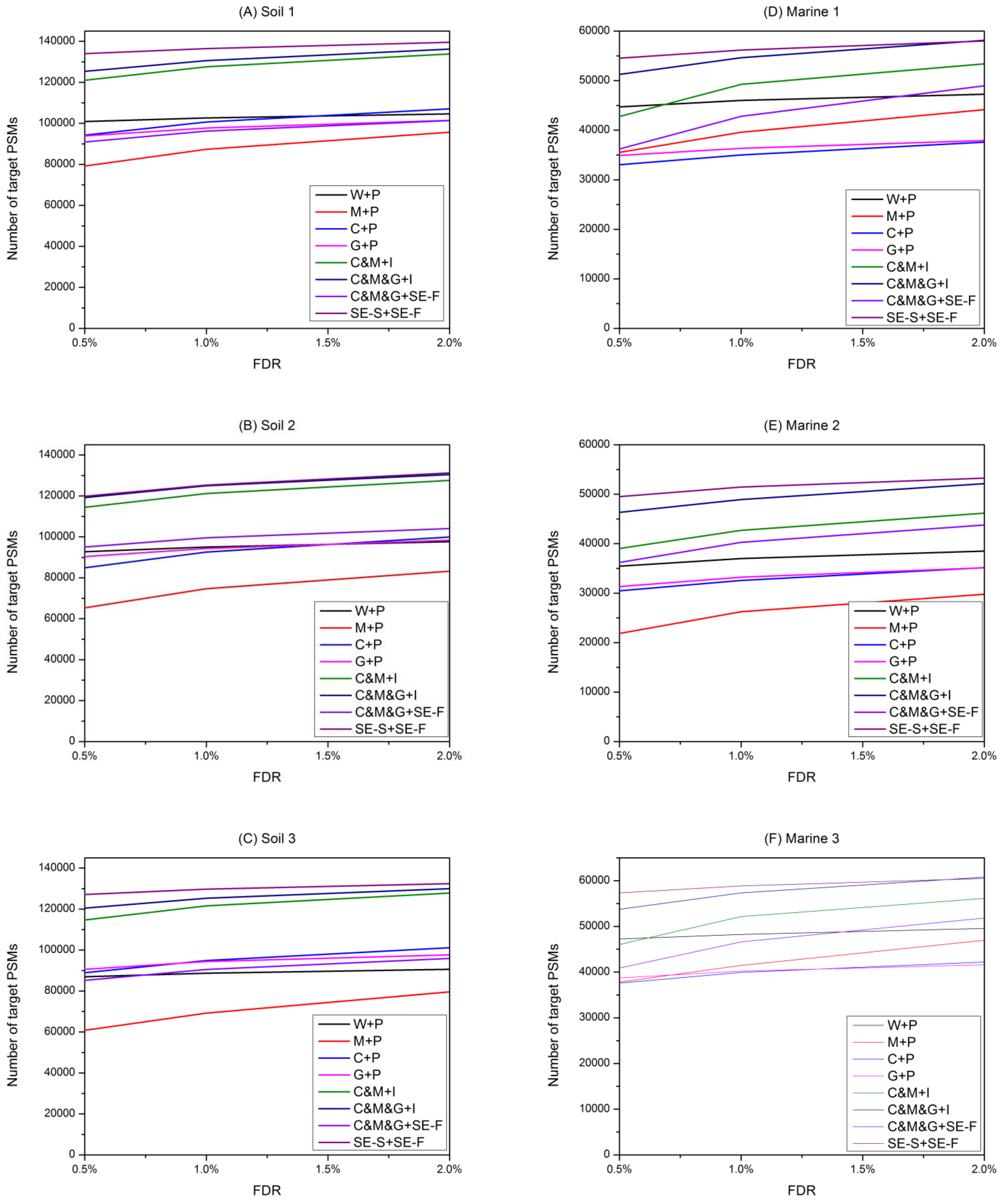

Supplementary Figure 3: Numbers of PSM identifications at PSM FDRs between 0.5% to 2%. The plots show the numbers of target PSMs as a function of PSM FDRs for soil 1 (A), soil 2 (B), soil 3 (C), marine 1 (D), marine 2 (E), and marine 3 (F). The different combinations of database searching and filtering algorithms are listed in the legend (SE-S: Sipros Ensemble Searching, SE-F: Sipros Ensemble Filtering, W: WDP, M: MyriMatch, C: Comet, G: MS-GF+, P: Percolator, I: iProphet).

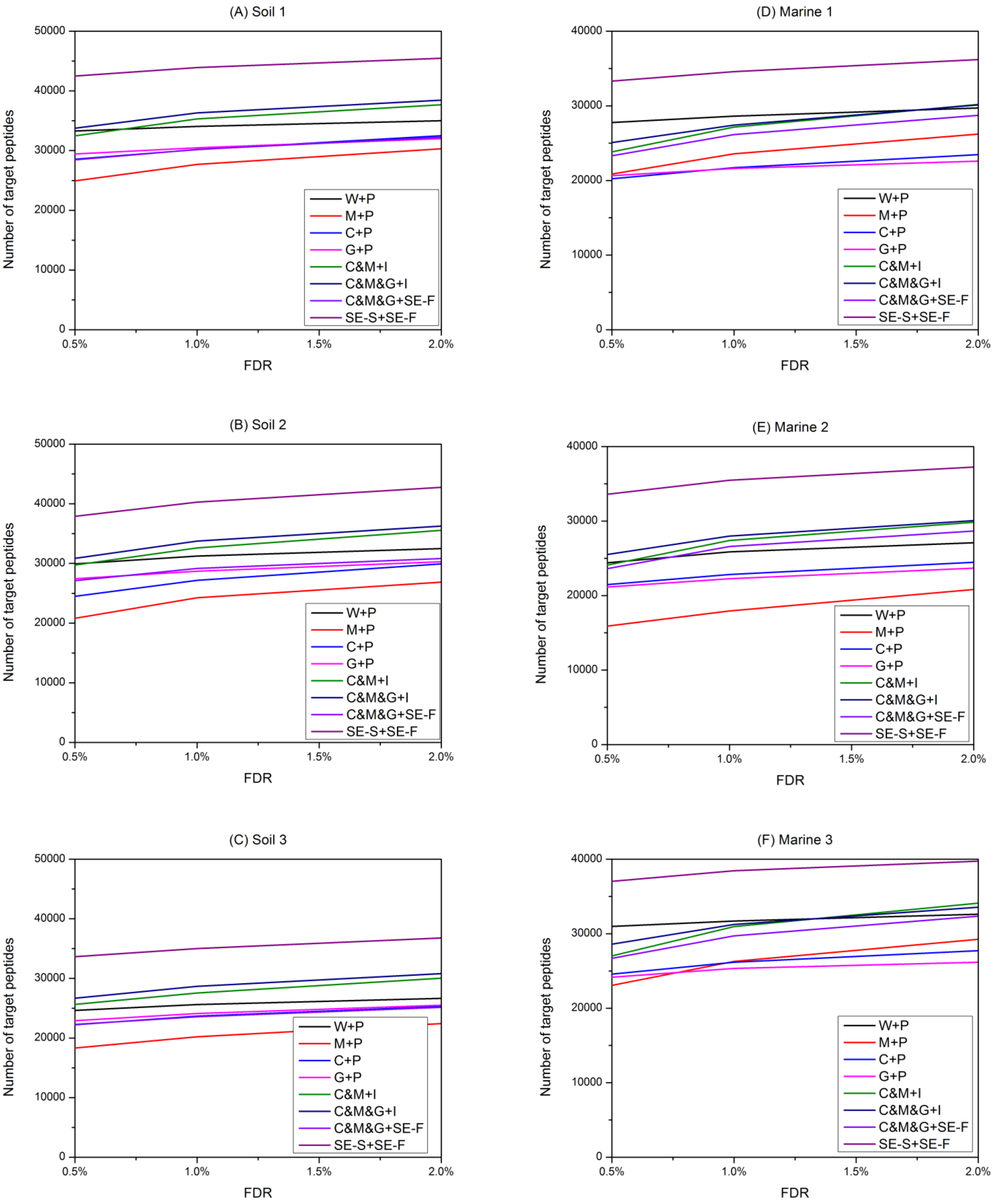

Supplementary Figure 4: Numbers of peptide identifications at peptide FDRs between 0.5% to 2%. The plots show the numbers of target peptides as a function of peptide FDRs for soil 1 (A), soil 2 (B), soil 3 (C), marine 1 (D), marine 2 (E), and marine 3 (F). The different combinations of database searching and filtering algorithms are listed in the legend (SE-S: Sipros Ensemble Searching, SE-F: Sipros Ensemble Filtering, W: WDP, M: MyriMatch, C: Comet, G: MS-GF+, P: Percolator, I: iProphet).

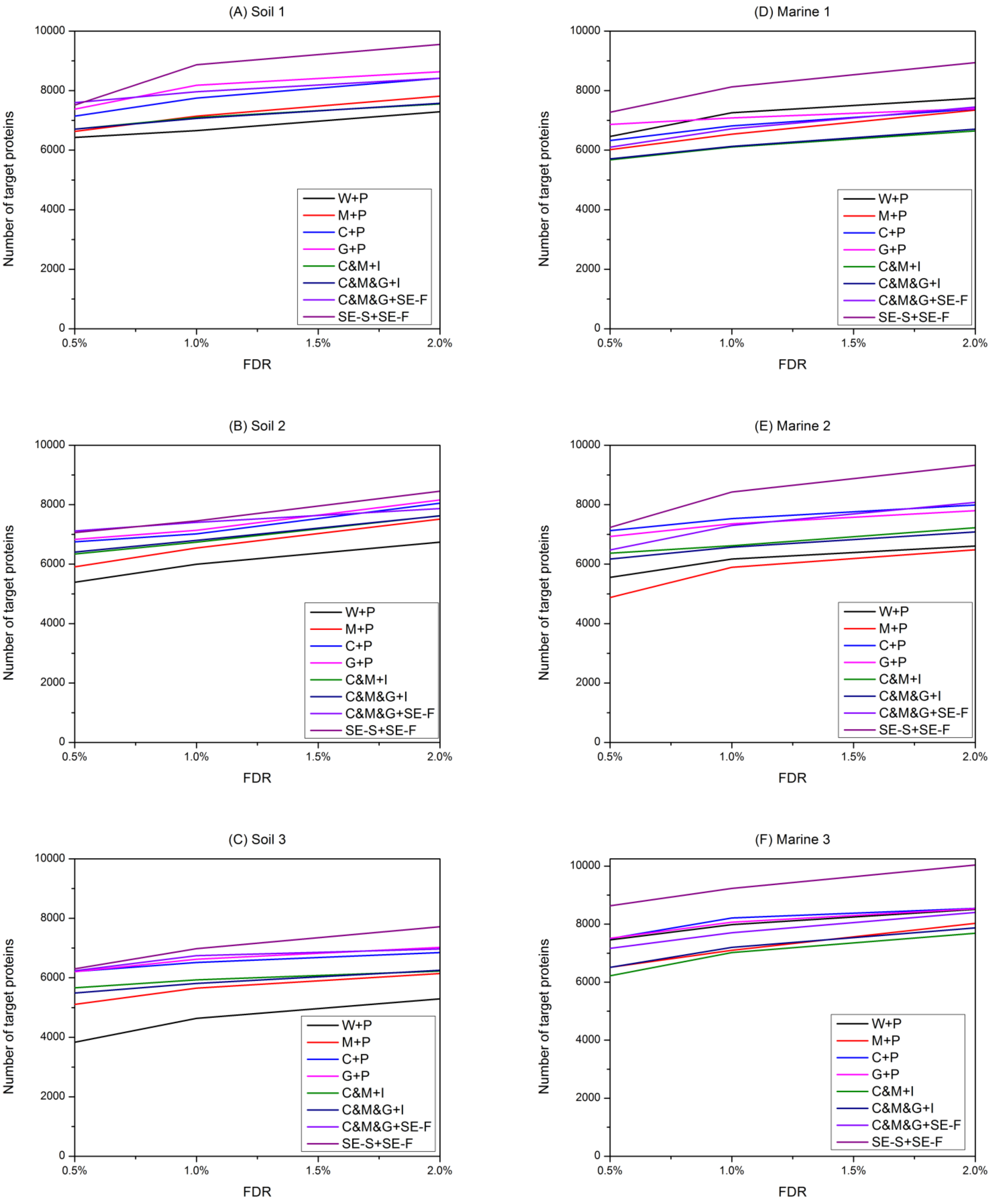

Supplementary Figure 5: Numbers of protein identifications at protein FDRs between 0.5% to 2%. The plots show the numbers of target proteins as a function of protein FDRs for soil 1 (A), soil 2 (B), soil 3 (C), marine 1 (D), marine 2 (E), and marine 3 (F). The different combinations of database searching and filtering algorithms are listed in the legend (SE-S: Sipros Ensemble Searching, SE-F: Sipros Ensemble Filtering, W: WDP, M: MyriMatch, C: Comet, G: MS-GF+, P: Percolator, I: iProphet).

Supplementary Table 1: The feature parameters of the four classifiers trained for two metaproteomes in Sipros Ensemble.

	Soil 1				Marine 1			
Features	Logistic Regression[1]	Random Forest[2]	AdaBoost[3]	Deep Learning[4]	Logistic Regression	Random Forest	AdaBoost	Deep Learning
MVH	0.009	0.170	0.085	0.007	0.005	0.114	0.115	0.045
Xcorr	0.007	0.057	0.065	0.057	-0.021	0.016	0.020	0.098
WDP	0.006	0.007	0.075	0.003	0.006	0.029	0.125	0.022
ΔMVH	0.039	0.091	0.155	0.037	0.044	0.133	0.140	0.123
ΔXcorr	0.375	0.073	0.100	0.120	0.752	0.090	0.090	0.149
ΔWDP	0.007	0.024	0.030	0.016	0.050	0.058	0.090	0.086
ΔM	0.016	0.001	0.035	0.312	0.043	0.001	0.080	0.190
#MCS	-0.073	0.006	0.010	0.038	-0.088	0.007	0.015	0.120
#PEP	-0.355	0.251	0.185	0.186	-0.400	0.291	0.165	0.018
#PRO	0.348	0.321	0.260	0.224	0.400	0.262	0.160	0.150

[1] Feature coefficients in logistic regression
[2] Feature importance in random forest
[3] Feature importance in AdaBoost
[4] Feature importance in neural networks.

Supplementary Table 2: Comparison of five supervised classification algorithms for PSM filtering at 1% FDR.

		Soil 1	Soil 2	Soil 3	Marine 1	Marine 2	Marine 3
Logistic Regression	# Target PSM	136,468	125,297	129,732	56,170	51,438	58,870
	Training FDR bias[1]	0.46%	0.40%	0.43%	0.10%	0.05%	0.17%
Random Forest	# Target PSM	125,588	119,452	118,164	54,846	50,514	57,397
	Training FDR bias	0.82%	0.79%	0.88%	0.82%	0.76%	0.85%
AdaBoost	# Target PSM	128,979	124,453	122,813	54,484	49,112	57,151
	Training FDR bias	0.82%	0.79%	0.85%	0.85%	0.76%	0.86%
Deep Learning	# Target PSM	132,143	130,240	129,168	52,629	48,190	55,363
	Training FDR bias	0.49%	0.46%	0.48%	0.22%	0.10%	0.31%
Stacking	# Target PSM	126,516	120,613	119,295	54,555	50,088	57,207
	Training FDR bias	0.86%	0.79%	0.85%	0.88%	0.85%	0.88%

[1]The FDR training biases were calculated as the differences between the training FDRs from the training set and the test FDRs from the test set.

Supplementary Table 3: PSM identifications from the four PSM classes at 1% PSM FDR.

		Soil 1	Soil 2	Soil 3	Marine 1	Marine 2	Marine 3
Unanimous PSMs	#[1]	101,254	88,722	88,358	41,003	35,656	44,251
	%[2]	80%	75%	81%	75%	75%	79%
Majority PSM: WDP & Xcorr	#	9,871	13,241	12,588	2,797	3,686	2,773
	%	23%	25%	29%	21%	24%	23%
Minority PSM: MVH	#	1,530	1,349	1,686	969	842	985
	%	4%	3%	4%	7%	10%	8%
Majority PSM: WDP & MVH	#	4,020	3,519	4,754	2,091	2,327	1,974
	%	16%	12%	20%	24%	28%	26%
Minority PSM: Xcorr	#	1,660	1,545	1,715	768	741	736
	%	6%	5%	7%	9%	9%	10%
Majority PSM: MVH & Xcorr	#	3,822	3,370	4,865	1,356	1,348	1,387
	%	19%	13%	21%	25%	18%	29%
Minority PSM: WDP	#	1,615	1,282	1,489	763	630	529
	%	8%	5%	6%	14%	8%	11%
Discordant PSM: WDP	#	2,874	2,817	2,703	2,302	2,065	2,155
	%	2%	1%	2%	5%	4%	6%
Discordant PSM: Xcorr	#	4,817	5,655	5,791	2,223	2,290	2,212
	%	3%	2%	4%	5%	4%	6%
Discordant PSM: MVH	#	5,005	5,079	5,783	1,900	1,853	1,868
	%	3%	2%	4%	4%	3%	5%
Total		136,468	125,297	129,732	56,170	51,438	58,870

[1]Numbers of PSMs passing the filtering

[2]Percentages of PSMs passing the filtering
