# Supplementary material for: Sipros Ensemble improves database searching and filtering for complex metaproteomics
Source: Bioinformatics. 2017 Sep 22;34(5):795–802. doi: 10.1093/bioinformatics/btx601 (PMC6192206; doi:10.1093/bioinformatics/btx601)
Supplement: Supplementary Data [file btx601_supp.zip › btx601-suppl_data/Sipros_Suppl_Methods_v7.pdf]

Supplementary Methods

Benchmarking data

A series of soil samples were collected from the Angelo Coast Range Reserve meadow (39° 44′ 21″ N 123° 37′ 51″ W) before and after two rains in the fall of 2013 and characterized by metagenomics and metaproteomics as described previously (Butterfield *et al.*, 2016). For benchmarking, we selected the representative soil samples collected from plot 1 at a depth of 30-40 cm on 08/20/2013 before the rains, plot 1 at a depth of 10-20 cm on 09/27/2013 six days after the first rainfall, and plot 1 at a depth of 30-40 cm on 10/02/2013 two days after the second rainfall. This study also used the matched metagenomes and metaproteomes of three marine communities collected from the coastal surface water of Monterey Bay, CA (36° 53’ 23″ N 121° 57’ 15″ W) on 10/14/2013 (Bryson *et al.*, 2016; Mueller *et al.*, 2015). For brevity, these metaproteomes were designated as soil 1, 2 and 3 and marine 1, 2 and 3 in this study. An *E. coli* proteome sample from a previous study (Li *et al.*, 2014) was also used here for benchmarking.

These (meta)proteome samples were analyzed using the Multidimensional Protein Identification Technology (MudPIT) approach (Washburn *et al.*, 2001) as described previously in the respective studies. Briefly, proteins from the soil samples were extracted using NoviPure® Soil Protein Extraction kit (Mo Bio) and concentrated using Amicon® Ultra-4 Centrifugal Filter Units (30 KDa molecular weight cut-off, Millipore). Proteins from the marine samples and the *E. coli* sample were extracted using sodium dodecyl sulfate lysis buffer. Extracted proteins from these samples were precipitated using trichloroacetic acid for overnight, washed using acetone, re-solubilized using 6M guanidine hydrochloride, and then reduced using 10 mM dithiothreitol. The protein samples were further processed via Filter-Aided Sample Preparation (Wiśniewski *et al.*, 2009) for proteolytic digestion with trypsin (Promega, product number V-5111). Using a pressure cell, peptide samples were loaded onto a bi-phasic sample column (or “back column”) constructed of a length of 150 µm i. d., 360 µm o. d. fused silica coupled to an in-line microfilter assembly (Upchurch/IDEX, M-520). Prior to sample loading into the back column, two lengths of stationary bulk media were packed into the fused silica tubing: first, 3 cm of strong cation exchange media (5 µm, 100 Å Luna SCX, Phenomenex) followed by 3 cm of reverse phase media (5 µm, 200 Å Aqua C18, Phenomenex), as described previously (McDonald *et al.*, 2002) [ref]. The back column was equilibrated with HPLC Solvent A (5% acetonitrile (CH_3CN), 95% H_2O, 0.1% formic acid (FA)) before and after peptide sample loading via pressure cell, then placed in-line with an analytical (or “front”) column containing a 15-cm length of C18 reverse phase media. A two dimensional liquid chromatographic separation was conducted at a flow rate of 500 nL/minute for a total of 22 hours. The two-dimensional separation was coupled to the nanosparay ionization source of an LTQ Orbitrap Elite mass spectrometer (Thermo Scientific). Tandem mass spectrometry data were recorded over 11 successive on-line SCX fractionation

using 5%, 7%, 10%, 12%, 15%, 17%, 20%, 25%, 35%, 50% and 100% of Solvent D (500 mM ammonium acetate in Solvent A) for each fraction, respectively. Peptides in each SCX fraction were separated by a 110-minute reverse phase gradient from 100% Solvent A to 50% Solvent B (70% CH_3CN, 30% H_2O, 0.1% FA). Both MS and MS/MS scans were acquired in Orbitrap using resolution of 30,000 and 15,000, respectively.

All mass spectrometry data and corresponding metaproteome databases have been deposited in public data repositories as described previously in the respective studies. Benchmarking data used in this study was consolidated and re-deposited into the ProteomeXchange Consortium via the PRIDE repository with the dataset identifiers of PXD007587. The filenames of the proteomics runs were Angelo_08202013_P1_3040cm_MB_FASP_Elite for soil 1, Angelo_09272013_P1_1020cm_MB_FASP_Elite for soil 2, Angelo_10022013_P1_3040cm_MB_FASP_Elite for soil 3, OSU_D10_FASP_Elite_03202014 for marine 1, OSU_D2_FASP_Elite_02262014 for marine 2, OSU_D7_FASP_Elite_03172014 for marine 3, and EColi_Try_HCD_DE10ppm_CS_1000_NCE30_180 for *E. coli*.

Protein databases were constructed from the metagenome assemblies of the matched environmental samples of the soil and marine communities (Bryson *et al.*, 2016; Butterfield *et al.*, 2016). There were 3,408,250 target proteins in the soil protein database and 391,847 target proteins in the marine protein database. The *E. coli* proteomics run was searched against a full *E. coli* protein database containing 4,146 proteins and two synthetic protein databases concatenating 50% randomly selected *E. coli* proteins to the full soil protein database for a total of 3,410,322 proteins and to a 10%-randomly-subsampled soil protein database for a total of 343,111 proteins. An equal number of reversed proteins were appended to the protein databases. All these benchmarking databases were also available in the PRIDE repository PXD007587.

Algorithm testing

Database searches were performed on the benchmarking datasets using Comet 2016.01 rev. 2, MyriMatch 2.2.8634, and MS-GF+ v2016.02.12, and Sipros Ensemble v1.0. The following parameters were specified for all database searching: precursor mass tolerance = 0.09 Dalton, fragment mass tolerance = 0.01 Dalton, full trypsin cleavage, the maximum number of missed cleavages = 3, peptide length from 7 to 60 residues, and dynamic modification of oxidation of methionine. All searches were executed on computer nodes equipped with Linux 2.6.32, a 16-core Intel Xeon E5-2650 CPU, and 128 Gb RAM. The scalability of Sipros Ensemble was tested on a high-performance computing system, Thunder, with SGI's Performance Suite and Linux 3.0.101-84 at the Air Force Research Laboratory (AFRL), Wright-Patterson Air Force Base, Dayton, OH.

The search results were filtered by Percolator v2.10, iProphet from TPP v5.0, and Sipros Ensemble v1.0 in default settings. We used the native support for the combinations of Comet searching with Percolator filtering, the combination of MS-GF+ searching with Percolator filtering, the combination of Comet and MyriMatch searching with iProphet filtering, and the combination of Comet, MyriMatch, and MS-GF+ searching with iProphet filtering.

Custom scripts were written for the following combinations of searching and filtering algorithms without native support. The features extracted from MyriMatch searching for Percolator filtering included mass difference, number of internal cleavage sites, experimental peptide mass, theoretical peptide mass, peptide charge, enzymatic cleavage terminals, MVH score, and MVH score differential. The features extracted from the WDP scoring results of Sipros Ensemble for Percolator filtering included mass difference, number of internal cleavage sites, experimental peptide mass, theoretical peptide mass, peptide charge, enzymatic cleavage terminals, WDP score, and WDP score differential. The following features were extracted for the combination of Comet, MyriMatch, and MS-GF+ searching with Sipros Ensemble filtering: EValue, EValue differential, MVH score, MVH score differential, Xcorr score, Xcorr score differential, mass difference, number of internal cleavage sites, spectrum count for the peptide of the PSM, and spectrum count for the protein or the protein group of the PSM.

These algorithms may estimate FDRs of their identifications using inconsistent methods internally from different subsets of the reversed proteins. To compare their results, one-third of the reversed proteins in a database were randomly selected and held out from all filtering algorithms by labeling them as target proteins. These held-out reversed proteins were used as the test set to estimate the test FDRs using Equation 4, in which the value of α was set to be 1/3 since one-third of the reverse proteins were used for FDR estimation. This provided an independent and consistent estimation of FDRs from the same subset of the reverse proteins across the different filtering algorithms. The remaining two-thirds of the reversed proteins were provided to Percolator, iProphet, and Sipros Ensemble. These filtering algorithms would use half of the provided reversed proteins for training and the other half for their internal FDR estimation. These algorithms were configured to output their filtering scores for all PSMs. PSM identification results were generated for each algorithm at the same test FDRs by filtering PSMs at a score threshold adjusted to reach the specified test FDRs at the PSM level.

The same rules were used to assemble peptide and protein identifications from the PSM identifications of all the tested searches. A peptide is identified if any of its PSMs is identified and a protein is identified if at least one unique peptide from this protein is identified. The peptide and protein identification results were generated for each search at the same test FDRs by filtering PSMs at a score threshold adjusted to reach the specified test FDRs at the peptide level or the protein level.

References

Bryson, S. *et al.* (2016). Proteomic Stable Isotope Probing Reveals Taxonomically Distinct Patterns in Amino Acid Assimilation by Coastal Marine Bacterioplankton. *mSystems*. Am Soc Microbiol **1**, e00027--15.

Butterfield, C. N. *et al.* (2016). Proteogenomic analyses indicate bacterial methylotrophy and archaeal heterotrophy are prevalent below the grass root zone. *PeerJ*. PeerJ Inc. **4**, e2687.

Li, Z. *et al.* (2014). Diverse and divergent protein post-translational modifications in two growth stages of a natural microbial community. *Nature Communications*. The Author(s) **5**, 4405.

McDonald, W. H. *et al.* (2002). Comparison of three directly coupled HPLC MS/MS strategies for identification of proteins from complex mixtures: single-dimension LC-MS/MS, 2-phase MudPIT, and 3-phase MudPIT. *International Journal of Mass Spectrometry*. Elsevier **219**, 245–251.

Mueller, R. S. *et al.* (2015). Metagenome sequencing of a coastal marine microbial community from Monterey Bay, California. *Genome announcements*. Am Soc Microbiol **3**, e00341--15.

Washburn, M. P. *et al.* (2001). Large-scale analysis of the yeast proteome by multidimensional protein identification technology. *Nature biotechnology*. Nature Publishing Group **19**, 242–247.

Wiśniewski, J. R. *et al.* (2009). Universal sample preparation method for proteome analysis. *Nature methods*. Nature Publishing Group **6**, 359–362.
