# Supplementary material for: Sipros Ensemble improves database searching and filtering for complex metaproteomics
Source: Bioinformatics. 2017 Sep 22;34(5):795–802. doi: 10.1093/bioinformatics/btx601 (PMC6192206; doi:10.1093/bioinformatics/btx601)
Supplement: Supplementary Data [file btx601_supp.zip › btx601-suppl_data/Sipros_Suppl_Results_v2.pdf]

Supplementary Results

Filtering of PSMs using retention time information

Previously, PSM retention times have been used to further filter the identification results of shotgun proteomics (Moruz *et al.*, 2010; Pfeifer *et al.*, 2007, 2009). This approach was also tested in Sipros Ensemble. The retention times of top-800 high-scoring target PSMs from a reversed-phase (RP) LC elution cycle were used to train two retention time prediction algorithms, RTPredict (Pfeifer *et al.*, 2007) and ELUDE (Moruz *et al.*, 2010). The two algorithms were then used to predict the retention times of both target and decoy PSMs filtered at 5% FDR by Sipros Ensemble. Figure S1 below compares the predicted and measured retention times of identified PSMs from an RP LC cycle of a soil sample. 10% of target PSMs (i.e. 326 PSMs) and 51% of decoy PSMs (i.e. 87 PSMs) had more than 20% deviation between their measured retention times and their predicted retention times by RTPredict (Figure S1A). Discarding these PSMs would reduce the PSM identification FDR from 4.7% to 2.5% at the expense of losing 10% of target PSMs. 6% of target PSMs (i.e. 219 PSMs) and 56% of decoy PSMs (i.e. 96 PSMs) had more than 20% deviation between their measured retention times and their predicted retention times by ELUDE (Figure S1B). Discarding these PSMs would reduce the PSM identification FDR from 4.7% to 2.2% at the expense of losing 6% of target PSMs. Although the additional filtering by retention time prediction reduced the FDRs of PSM identification without a significant loss of target PSMs, this operation was not incorporated in Sipros Ensemble to simplify its computational workflow

Figure S1: Comparison of predicted and measured retention times of PSMs identified at 5% FDR. The black circles represent target PSMs. The red crosses represents decoy PSMs. (A) Predicted retention times of PSMs from RTPredict. (B) Predicted retention times of PSMs from

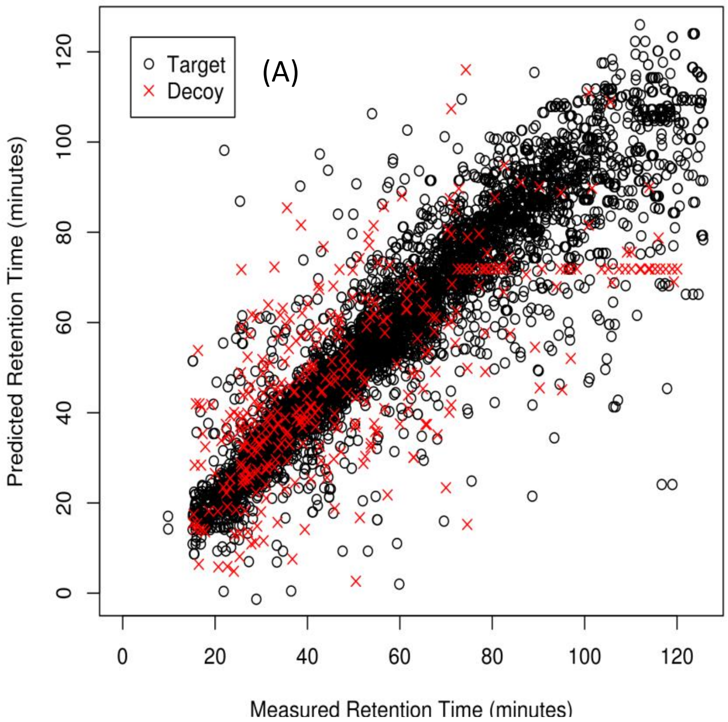

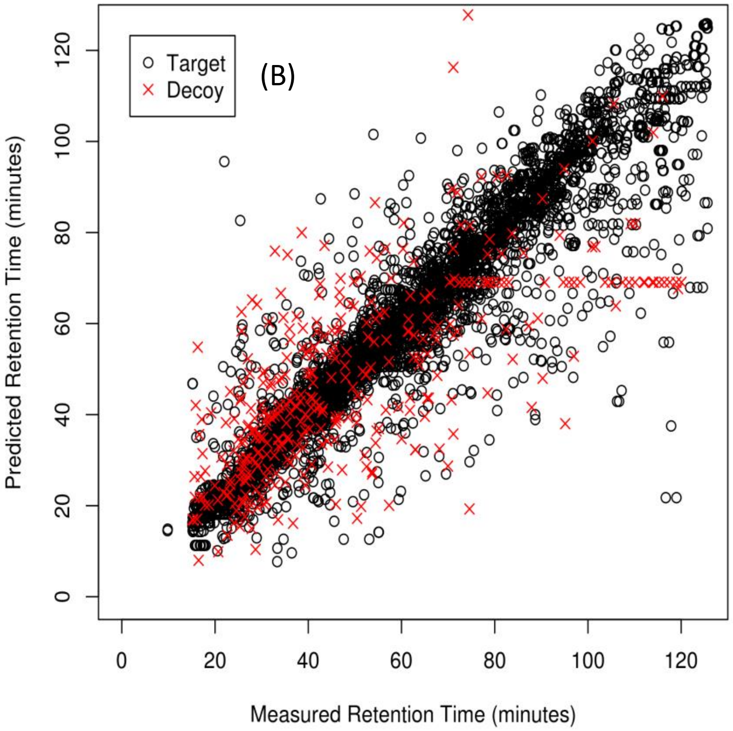

References

Moruz, L. *et al.* (2010). Training, selection, and robust calibration of retention time models for targeted proteomics. *Journal of proteome research*. ACS Publications **9**, 5209–5216.

Pfeifer, N. *et al.* (2007). Statistical learning of peptide retention behavior in chromatographic separations: a new kernel-based approach for computational proteomics. *BMC bioinformatics*. BioMed Central **8**, 468.

Pfeifer, N. *et al.* (2009). Improving peptide identification in proteome analysis by a two-dimensional retention time filtering approach. *Journal of proteome research*. ACS Publications **8**, 4109–4115.
